# Topics of Public Interest

**Published:** 1911-06

**Authors:** 


### TOPICS OF PUBLIC INTEREST

### Alcohol for Hand Disinfection

According to the observations of Professor Schumburg a surgeon on the general staff of the German Army reported in the *Deutsche Medizinische Wochenschrift* washing the hands with strong alcohol is a most effective means of removing all infection and rendering any bacteria innocuous. This author states that 200 c.cm. of alcohol applied with a pledge of cottonwool are sufficient to disinfect the hands to the extent of 99 per cent. or more of all bacteria present. Ordinary methylated spirit is quite effective. From experiments in the medical department of the Prussian Ministry of War it appears that washing with soap and water combined with even prolonged scrubbing with a brush does not remove the microbes, the soap softening the skin and making the bacteria more adherent. Alcohol, on the other hand, by hardening the skin, causes the bacteria to become rapidly detached. To secure proper disinfection with alcohol the preliminary use of soap and water must be dispensed with, the reasons given being that the residual moisture, even after drying, dilutes the alcohol, and further that the softening of the skin by water causes it to contract too strongly when the alcohol is applied, and by rendering it rough and scaly encourages the transference of bacteria from the surgeon's hands to the wound. Inasmuch, however, as the wearing of gloves for operations has been now so generally adopted, disinfection of the hands has not the same importance that it once possessed for the success of aseptic surgery.

### The Fight Against Tuberculosis

In an encouraging letter to 1,500 officers of local antituberculosis committees in this state affiliated with the State Charities Aid Association, Ex-Ambassador Joseph H. Choate, president of that association, summarises the progress which has been made in one year toward the realisation of "No uncared-for tuberculosis in New York state in 1915." President Choate points out that the number of county hospitals authorised has increased in a year from 8 to 16; the number of dispensaries from 15 to 25; the number of visiting nurses from 32 to 39; while the localities providing special relief has increased from 8 to 15.

In the course of his letter President Choate says: "The following table showing the agencies in the State, outside of New York

City, devoted to the prevention of tuberculosis on October 1, 1907, when our work was begun; on March 1, 1910, just before the notable Albany Conference at which President Taft spoke; and on this date, April 27, 1911, states in the briefest possible space the results up to the time of our three years' work:

1. State hospital beds, Oct. 1, 1907, 164; Mar. 1, 1910, 328; Apr. 27, 1911, 328.
2. County hospitals definitely assured, Oct. 1, 1907, 0; Mar. 1, 1910, 8; Apr. 27, 1911, 16.
3. City hospitals definitely assured, Oct. 1, 1906, 1; Mar. 1, 1910, 11; Apr. 27, 1911, 12.
4. Camps, Oct. 1, 1907, 0; Mar. 1, 1910, 7; Apr. 27, 1911, 7.
5. Free dispensaries and relief stations, Oct. 1, 1907, 2; Mar. 1, 1910, 15; Apr. 27, 1911, 25.
6. Visiting nurses, Oct. 1, 1907, 2; Mar. 1, 1910, 32; Apr. 27, 1911, 39.
7. Localities providing special relief, Oct. 1, 1907, 2; Mar. 1, 1910, 8; Apr. 27, 1911, 15.

---

### Open Air Schools for Children

SINCE January 1, 1907, sixty-five open air schools for children afflicted with or predisposed to tuberculosis have been established in twenty-eight cities, according to an announcement made in a bulletin issued recently by the National Association for the Study and Prevention of Tuberculosis.

The first open air school in the United States was established on January 1, 1907, by the board of education of Providence, R. I. The next school was established in May of the same year at Pittsburg, a third one at Boston in July, 1908, and the fourth at Bellevue Hospital in New York in December, 1908. During the year 1909 ten schools in five different cities were opened; in 1910, sixteen schools in twelve cities were opened; and eight schools in five cities have been opened to April 1, 1911, while definite provision has been made for twenty-seven more schools in six cities. Many cities are considering the question and will act during the coming year.

New York now has in operation twelve open air schools, and definite provision has been made for fourteen similar classes to be opened by next fall. Boston has five open air classes in its schools, and Chicago has several.

According to reports received by the National Association, the result of the open air class-work has been to restore most of the children to normal health and efficiency. One of these open air schools or classes should be established for each 25,000 population, especially in cities.

